# Supplementary material for: Nsun4 and Mettl3 mediated translational reprogramming of Sox9 promotes BMSC chondrogenic differentiation
Source: Commun Biol. 2022 May 25;5:495. doi: 10.1038/s42003-022-03420-x (PMC9133052; doi:10.1038/s42003-022-03420-x)
Supplement: Supplementary file 2 — Supplementary Information [file 42003_2022_3420_MOESM2_ESM.pdf]

Nsun4 and Mettl3 mediated translational reprogramming of Sox9 promotes BMSC chondrogenic differentiation

Supplementary Figures

Supplementary Figure 1. The m^5C and m^6A levels during chondrogenic differentiation.

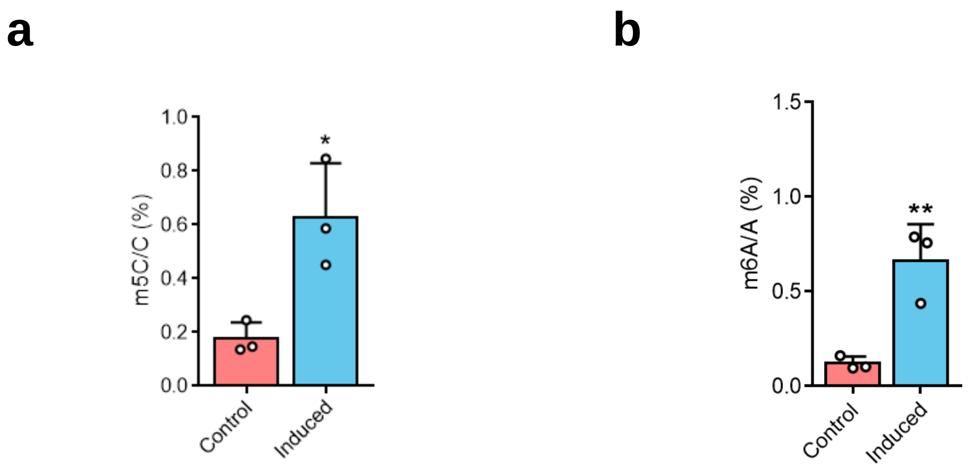

a, b The m^5C/C ratio **a** and m^6A/A ratio **b** of the mRNA were determined by LC-MS/MS after chondrogenic differentiation. Data are presented as means ± SD from three independent experiment. * $P < 0.05$, ** $p < 0.01$, by Student's t test.

Supplementary Figure 2. The role of Nsun4 and Mettl3 during chondrogenic differentiation.

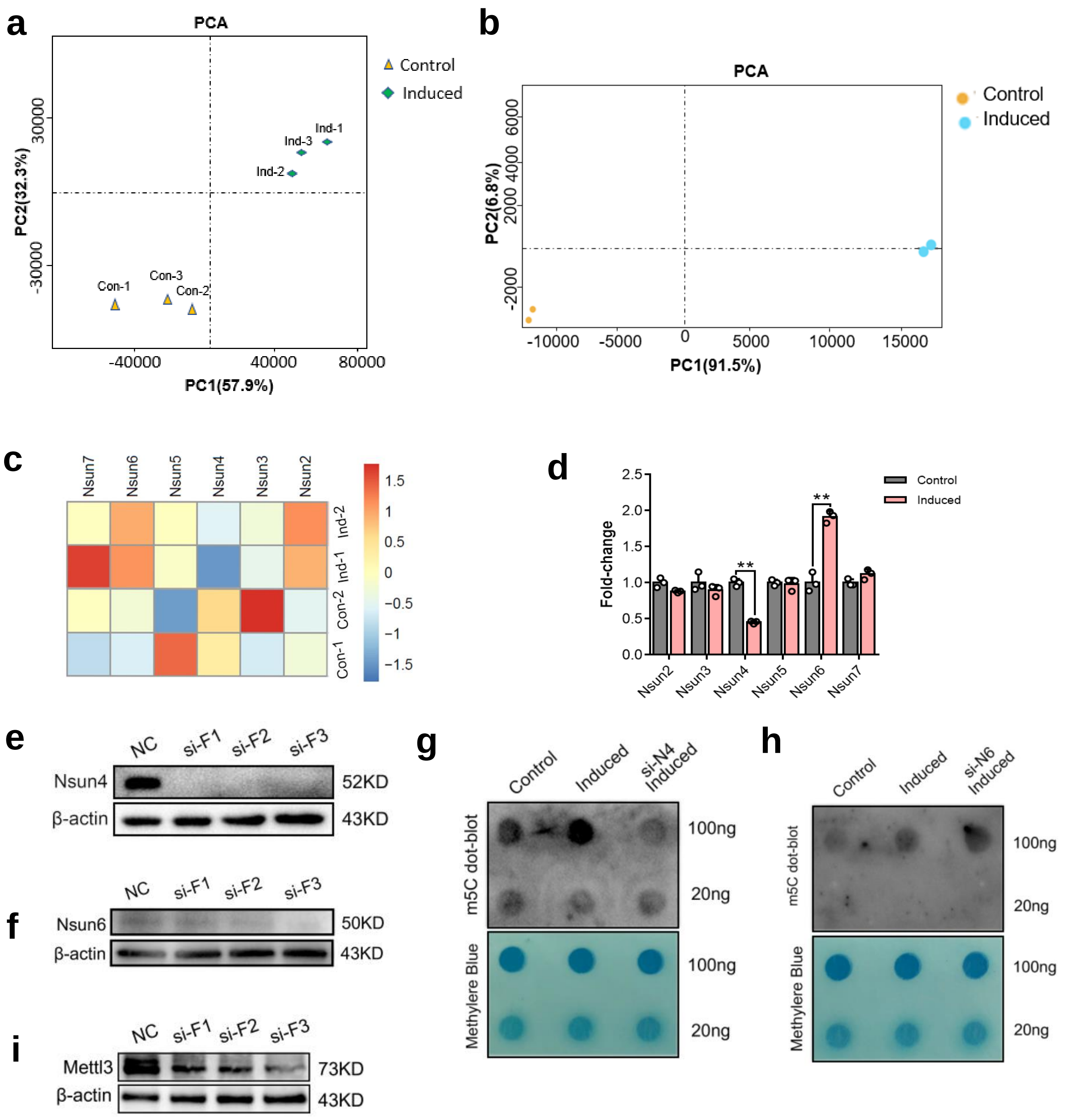

a, b Principal component analysis (PCA) of ribosome profiling (**a)** and RNA-sequencing (**b)** libraries from the control cells and BMSCs cultured in chondrogenic induce medium for 7 days (n=3 for ribosome profiling and n=2 for RNA-sequencing. **c** Nsun2-7 mRNAs were detected by RNA-seq. Heat maps show the differential expression fold(log$_2$Rario) for Nsun2-7 mRNAs between control and induced group. **d** NSUN2-7 mRNA expression on chondrogenic differentiation tested by RT-qPCR. **e, f , i** Western blot analysis of Nsun4 (**e**), Nsun6 (**f)** and Mettl3 (**i)** in BMSCs transfected with the indicated siRNAs. **g, h** m^{5}C dot blot of the Nsun4 knockdown (**g)** and Nsun6 knockdown (**h)** BMSCs undergoing chondrogenic differentiation. Data are presented as means ± SD from three independent experiment. * $P < 0.05$, **$p < 0.01$, ***$p < 0.001$ compared with the control group by Student's t test.

Supplementary Figure 3. Nsun4 and Mettl3 stable each other during chondrogenic differentiation .

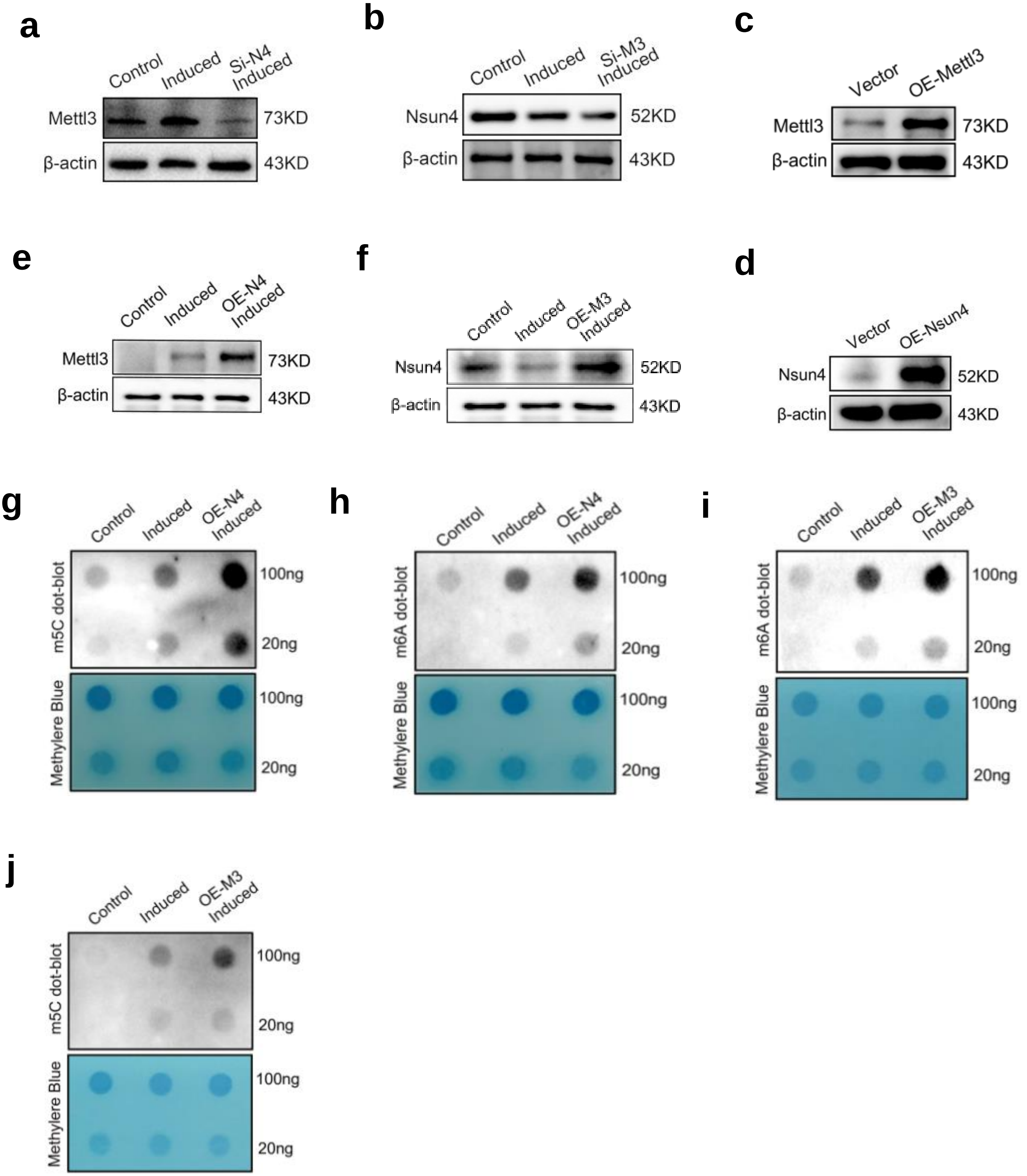

a, e Protein expressions of Mettl3 in Nsun4 knockdown (**a**) and overexpressed (**e**) BMSCs after 7 days of chondrogenic induction. **b, f** Protein expressions of Nsun4 in Mettl3 knockdown (**b**) and overexpressed (**f**) BMSCs after 7 days of chondrogenic induction. **c, d** Verification of the efficiency of Mettl3 or Nsun4-carrying adenovirus (OE-Mettl3 or OE-Nsun4) to induce Mettl3 (**c**) or Nsun4 (**d**) overexpression at the protein level after adenovirus infection in BMSCs. **g-j** Dot blot analysis of m^5C (**g, j**) and m^6A (**h, i**) in total mRNA of the BMSCs infected with adenovirus of Mettl3 or Nsun4 after induction for 7days.

Supplementary Figure 4. The efficiency of RNA bisulfite conversion treatment.

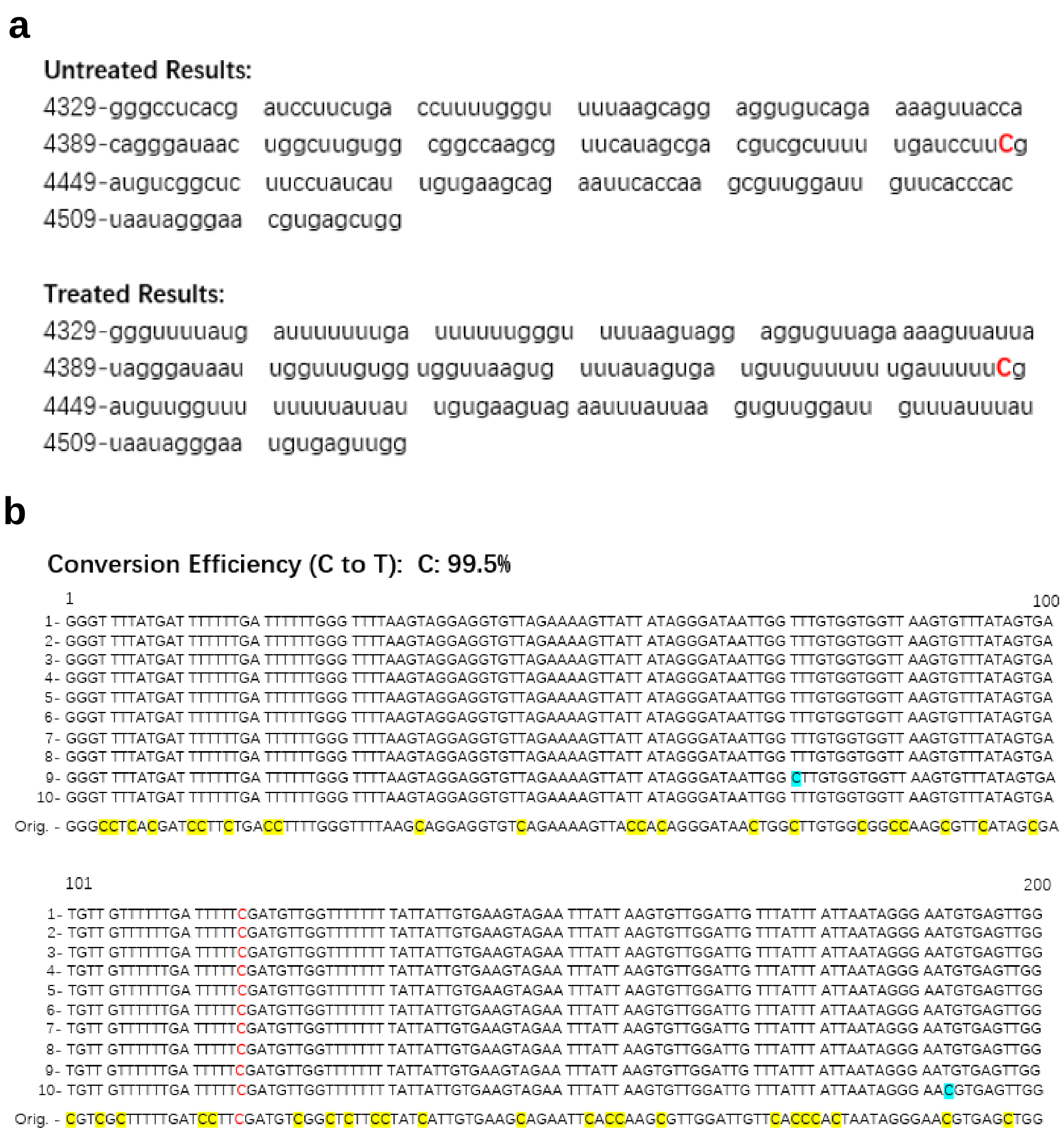

a m[5]C sites in 28S rRNA identified by RNA-BisSeq. **b** The conversion rate of RNA-BisSeq. Highlighted C represents m[5]C, highlighted C represents non-converted cytosine. The original, non-converted RNA sequence with non-methylated C highlighted is show below the converted cDNA sequencing results for comparison.

Supplementary Figure 5. Identification of m5C and m6A sites in Sox9 3‘UTR.

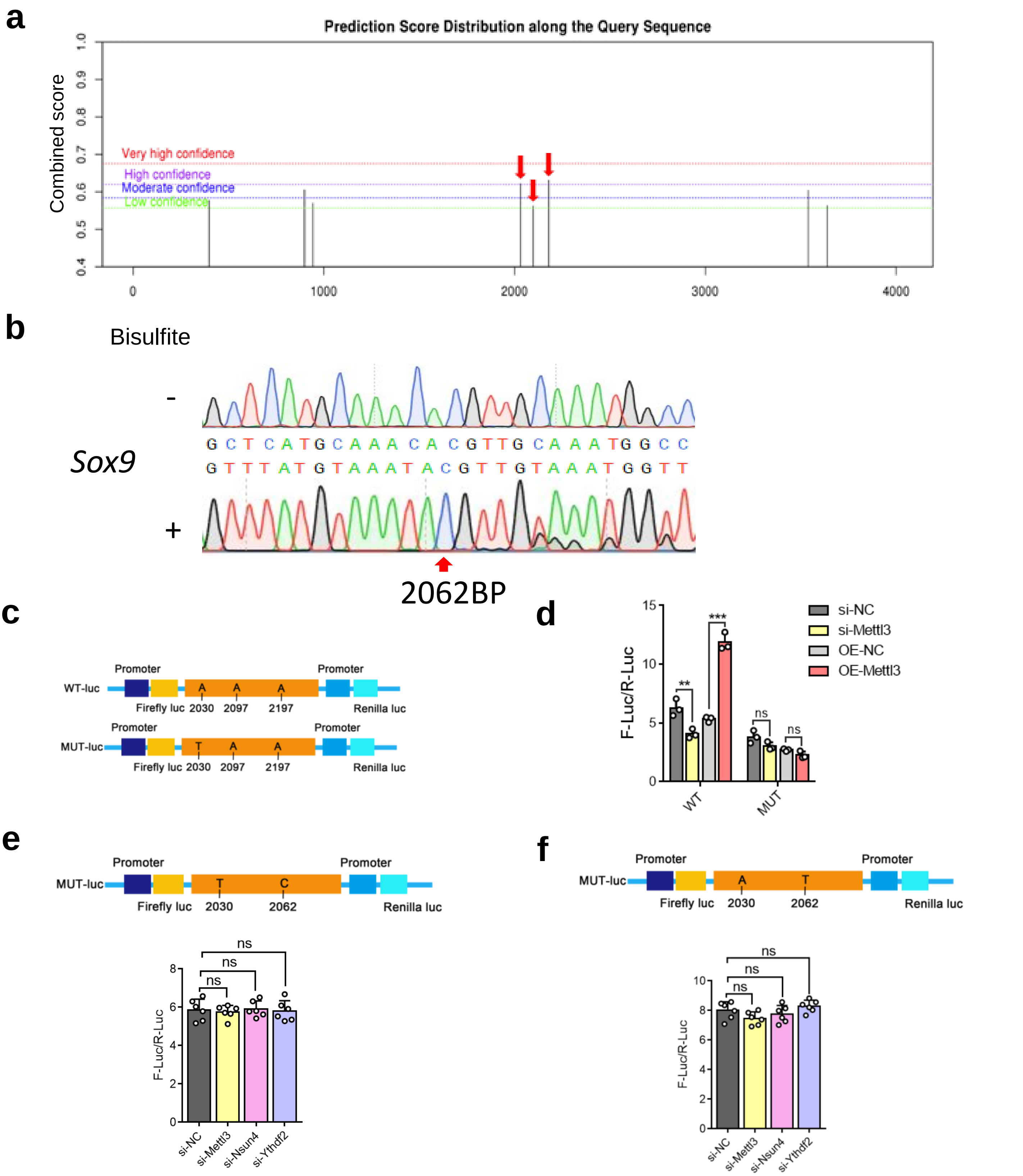

a Predicted sites for m^{6}A modification in the sequence of *Sox9* gene. **b** m^{5}C sites in 3’UTR of *Sox9* identified by RNA-BisSeq. **c** Schematic depiction of Sox9 3’UTR wide type (WT-luc) and mut type (MUT-luc) reporters. For the mut type (MUT-luc), A to T was made in the 2030nt. **d** Relative dual-luciferase reporter activity of wide (WT-luc) or mutated (MUT-luc) reporters in 293T cells with knock-down and overexpressed Mettl3. **e, f** Schematic diagram of *Sox9* 3’UTR MUT-luc reporters. The MUT-luc, A or C to T substitutions were made (up). Relative dual-luciferase reporter activity of Mut-luc reporter in 293T cells with ectopically expressed Mettl3, Nsun4 and Ythdf2 respectively (down). Data are presented as means ± SD from three or six independent experiment. * $P < 0.05$, ** $p < 0.01$, *** $p < 0.001$ compared with the negative control group by one way ANOVA and Tukey’s multiple comparison tests .

Supplementary Figure 6. The proteins interacted with Nsun4.

a

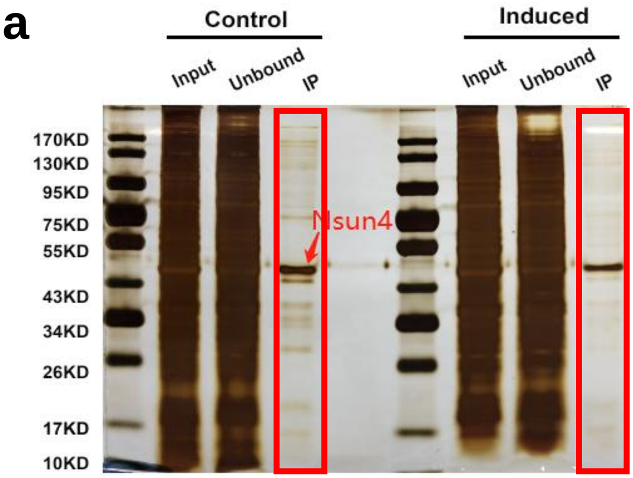

b

A list of indentified Nsun4-interaction protein candidates

Detected specially in BMSCs on chondrogenic differentiation	
Gene names	Major function
RPS17	translation
RPS25	translation
RPS30	translation
RPL39	translation
Rpl10a	translation
EF1A1	mRNA binding; translation elongation factor activity
Mettl3	mRNA (N6-adenosine)-methyltransferase activity; mRNA binding

a Sliver-stained gel of affinity-purified Nsun4 complex in BMSCs undergoing chondrogenic differentiation. **b** The table of a list of protein candidates that interacted with Nsun4 protein.

Supplementary Figure 7. Protein expression and purification.

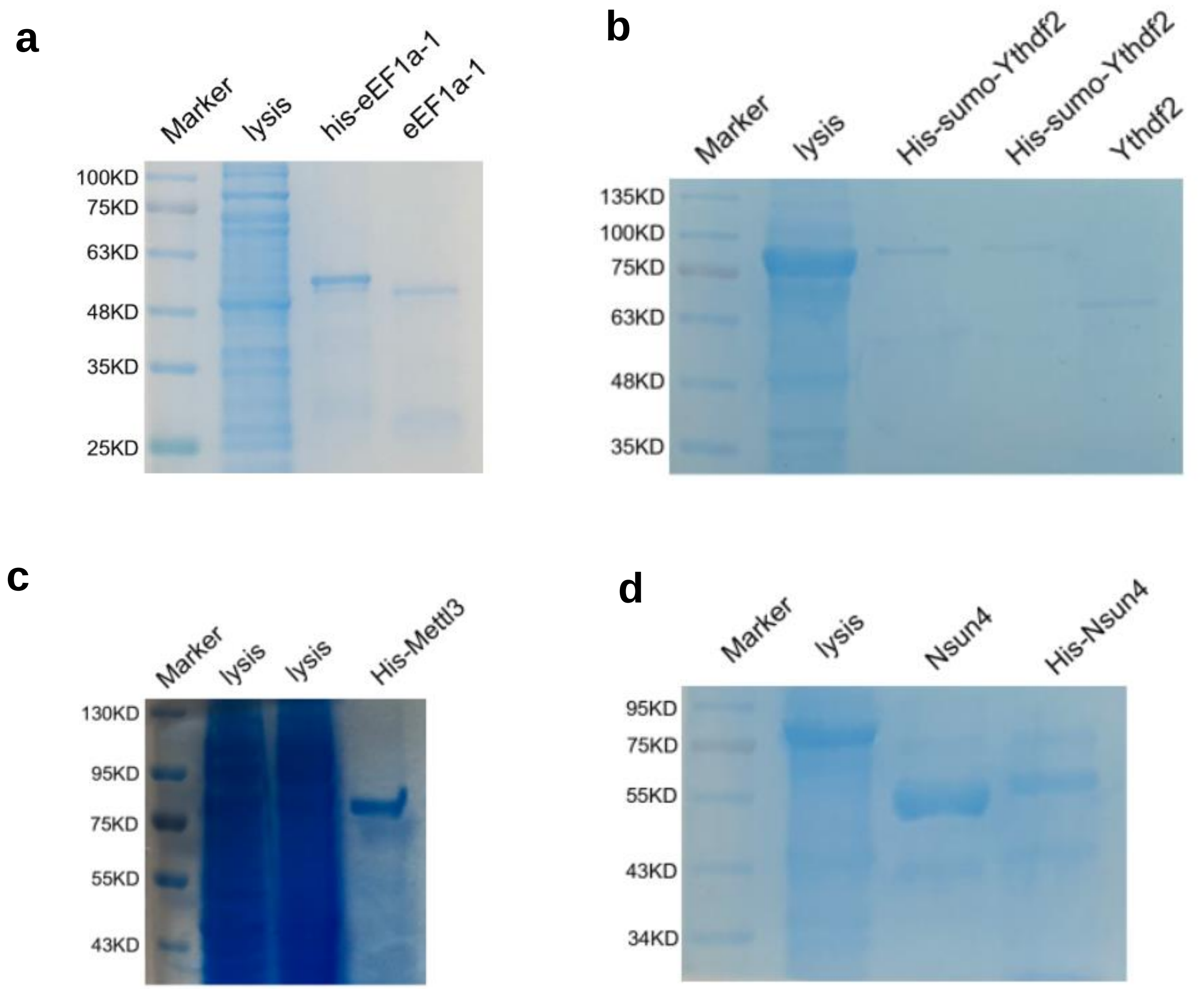

a-d SDS-PAGE analysis of the eEF1α-1, Ythdf2, Mettl3 and Nsun4.

Supplementary Figure 7. The expression of marker genes regulated by Mettl3 and Nsun4.

a

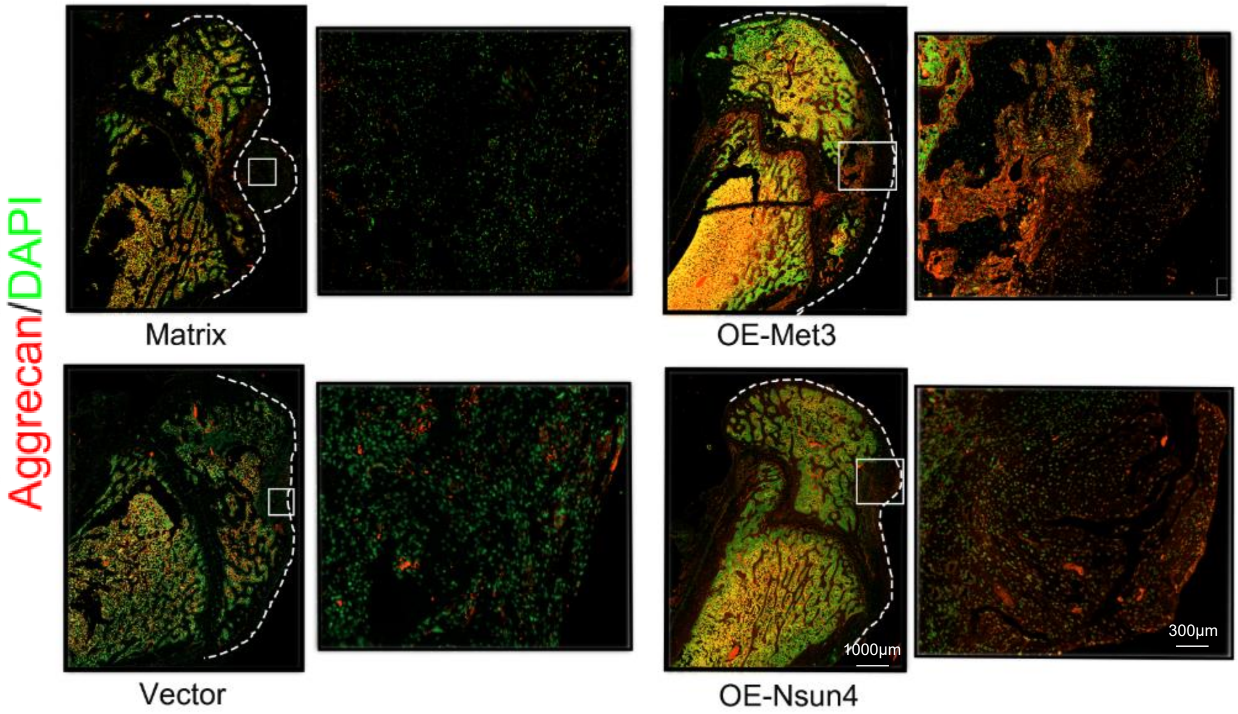

b

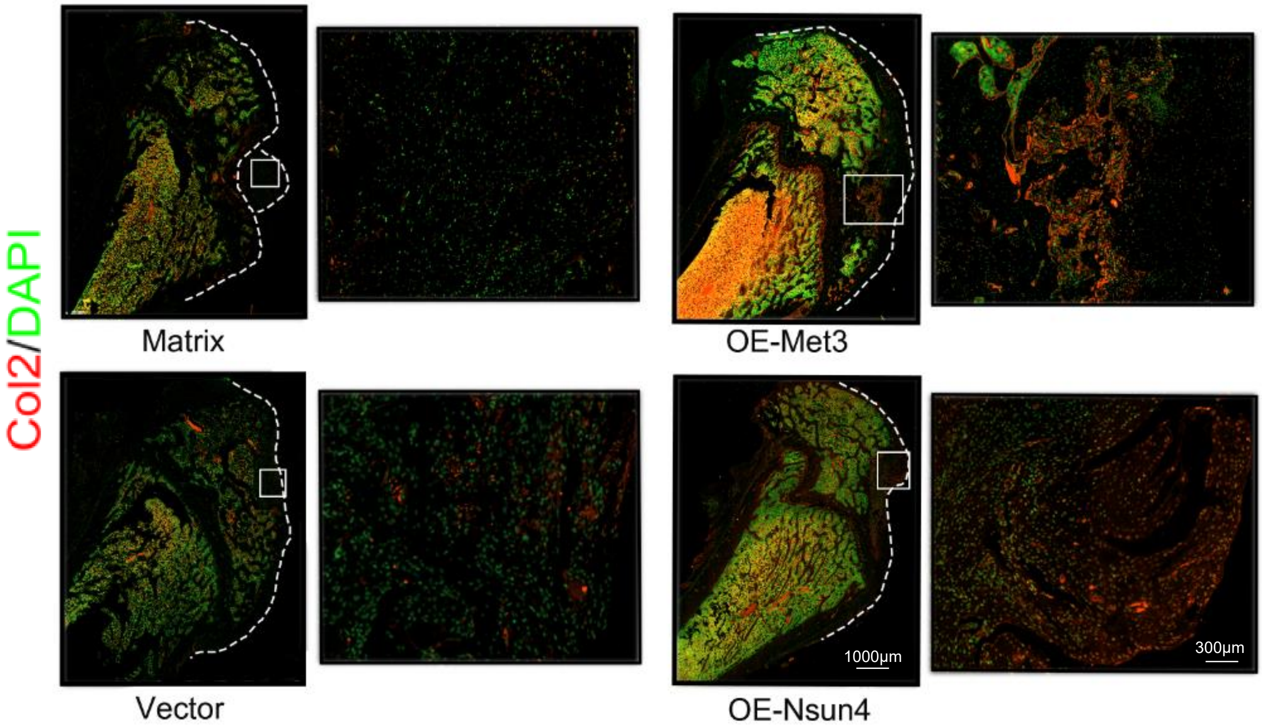

a, b Aggrecan (**a**) and Col2 (**b**) protein expression of repaired cartilage visualized through immunofluorescence at six weeks.

Original Blot Scans – Main Article

Original scans Figure 1

Figure 1b

Sox9:

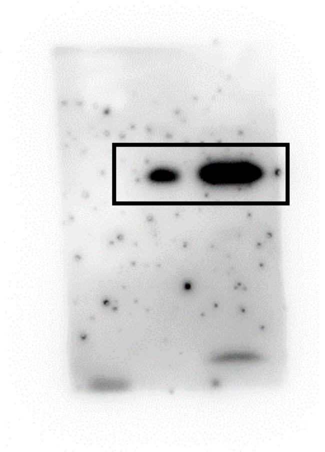

Aggrecan:

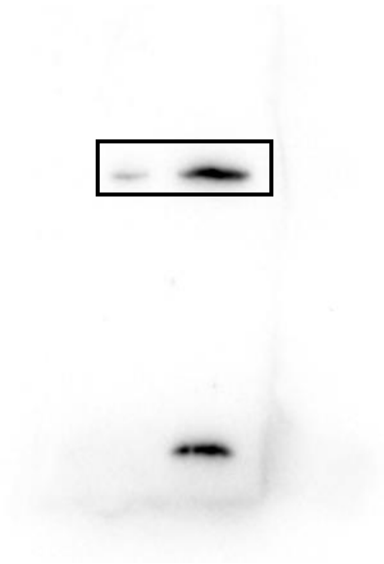

Col2:

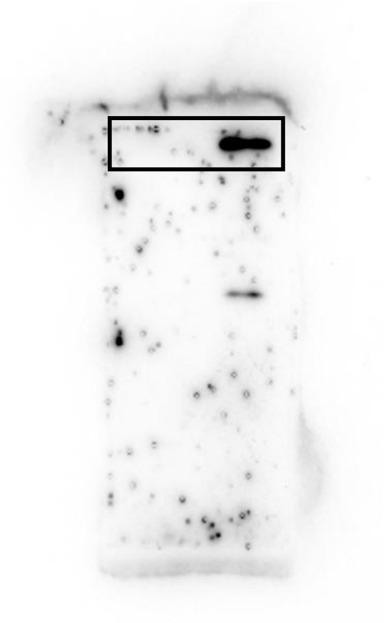

β-actin:

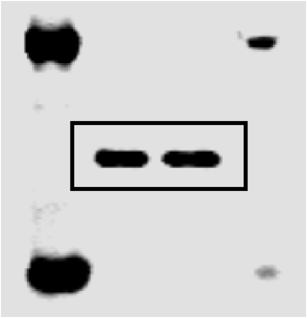

Figure 1e

m^5C:

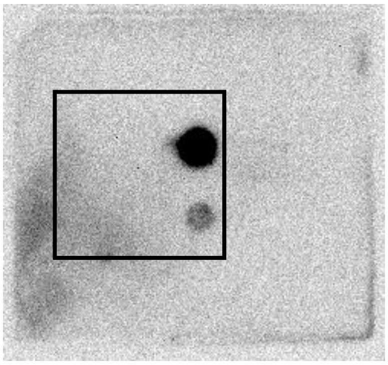

Figure 1f

m^6A:

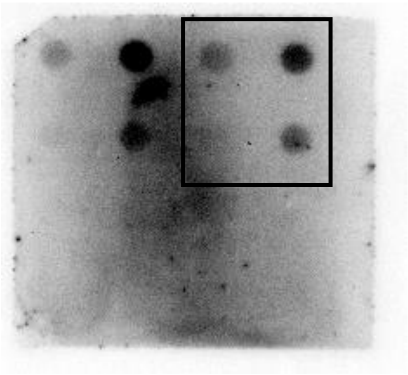

Original scans Figure 2

Figure 2a:

Nsun4:

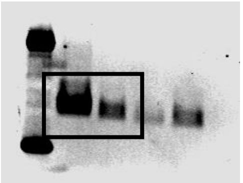

β-actin:

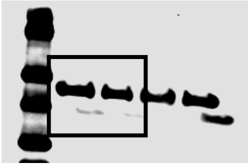

Figure 2b:

Mettl3:

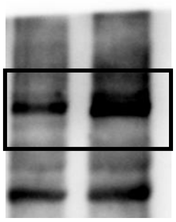

β-actin:

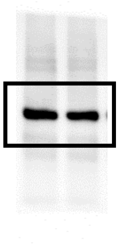

Figure 2f:

Sox9:

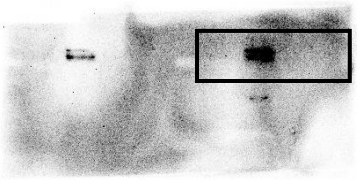

Aggrecan:

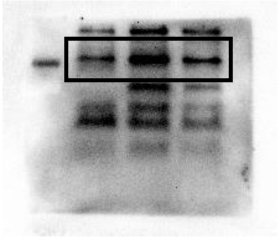

Col2:

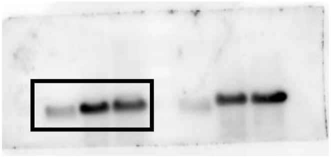

β-actin:

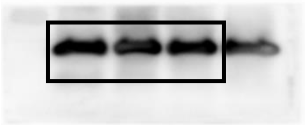

Figure 2g:

Sox9:

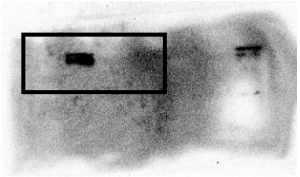

Aggrecan:

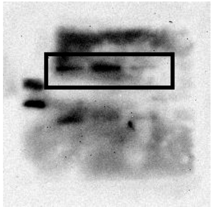

Col2:

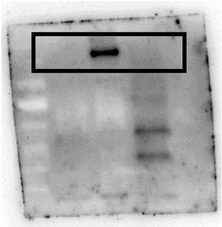

β-actin:

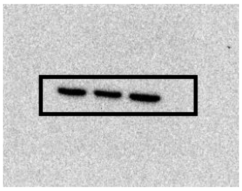

Figure 2h:

m^5C:

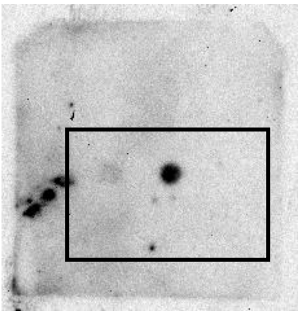

Original scans Figure 3

Figure 3a:

IP-Mettl3:

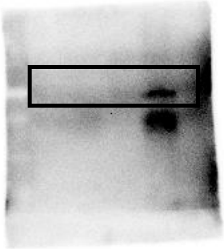

Input-Mettl3:

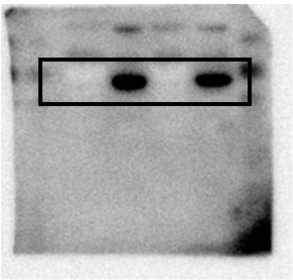

Input-Nsun4:

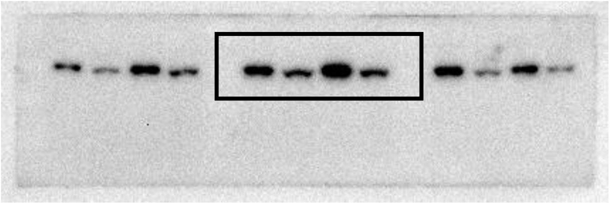

Input- β-actin :

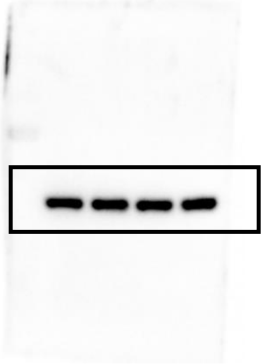

Figure 3b:

IP-Nsun4:

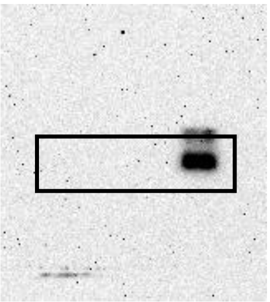

Input-Mettl3:

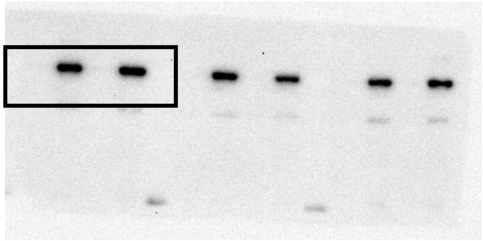

Input-Nsun4:

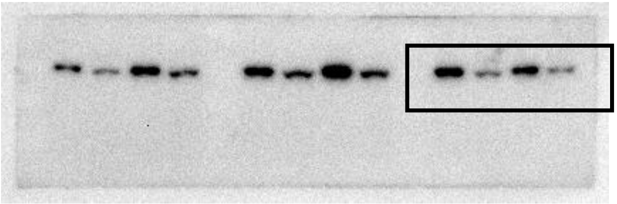

Input- β-actin :

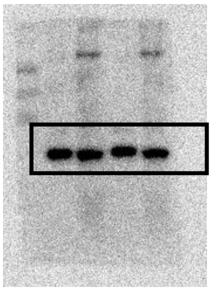

Figure 3c:

m^5C:

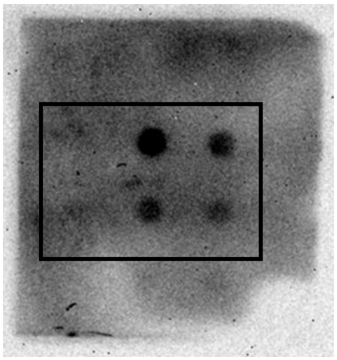

Figure 3d:

m^6A:

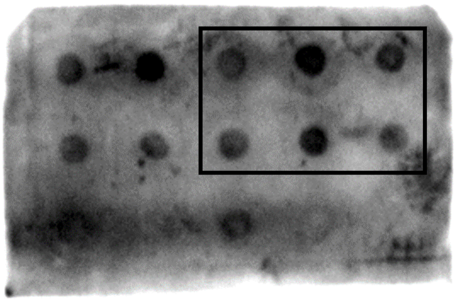

Original scans Figure 4

Figure 4a:

IP-eEF1a-1:

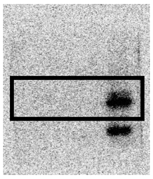

Input-Mettl3:

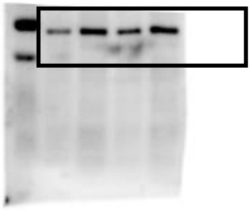

Input-eEF1a-1:

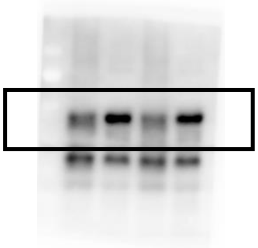

Input- β-actin :

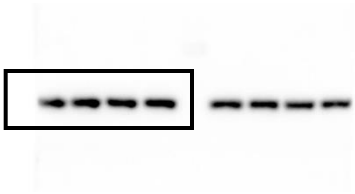

Figure 4b:

IP-eEF1a-1:

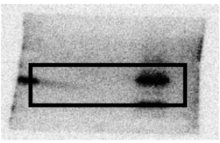

Input-eEF1a-1:

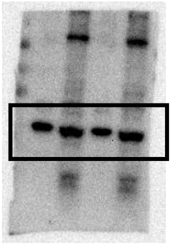

Input-Nsun4:

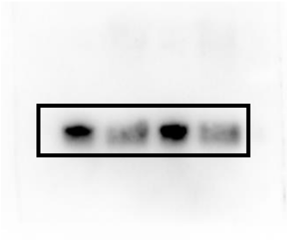

Input- β-actin :

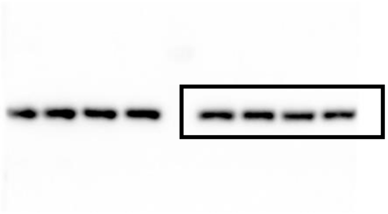

Figure 4c:

IP-Ythdf1:

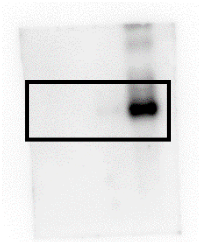

IP-Ythdf2:

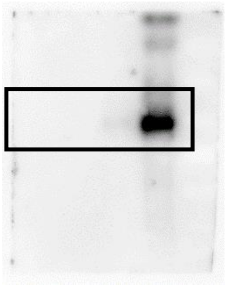

IP-Ythdf3:

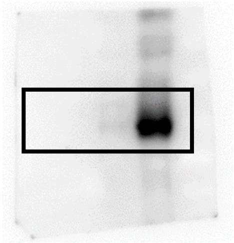

Input-Ythdf1:

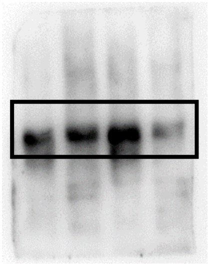

Input-Ythdf2:

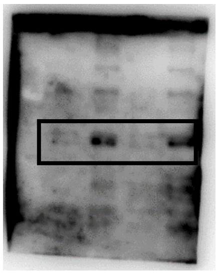

Input-Ythdf3:

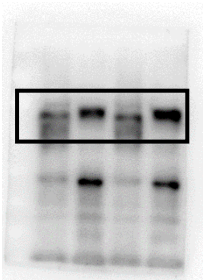

Input-Mettl3:

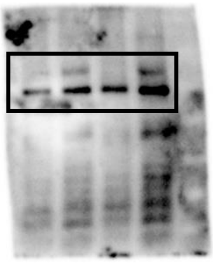

Input- β-actin :

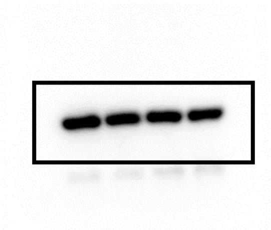

Figure 4d:

IP-Ythdf2:

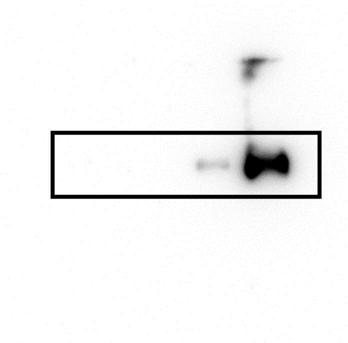

Input-Ythdf1:

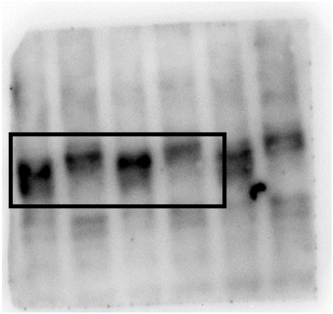

Input-Ythdf2:

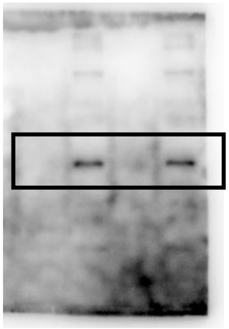

Input-Ythdf3:

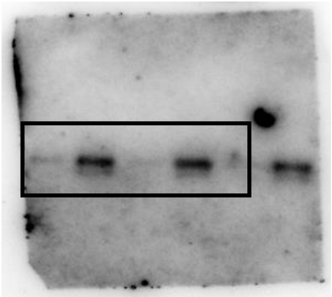

Input-Nsun4:

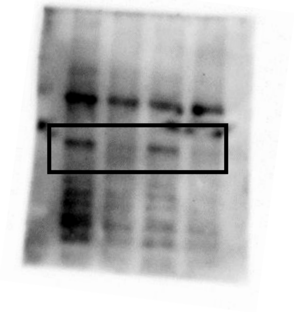

Input- β-actin :

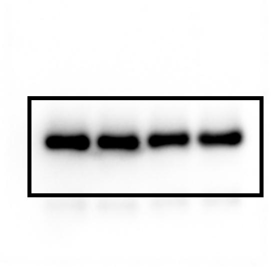

Figure 4e:

Sox9:

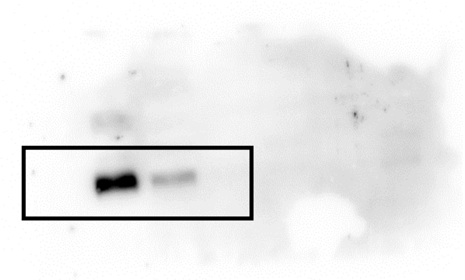

β-actin:

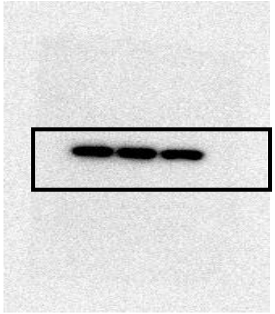

Figure 4e

Mettl3:

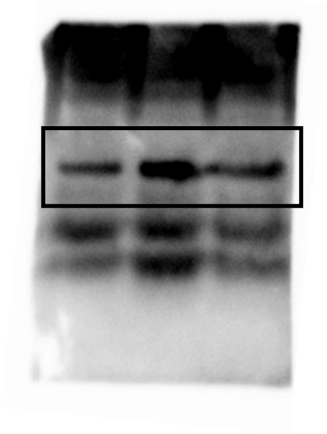

Nsun4

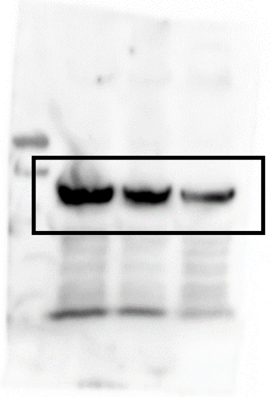

B-actin:

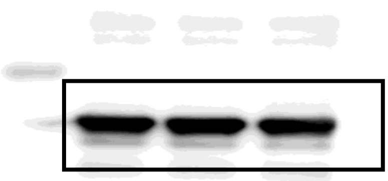

Original scans Supplementary Figure 1

Supplementary Figure 1e:

Nsun4:

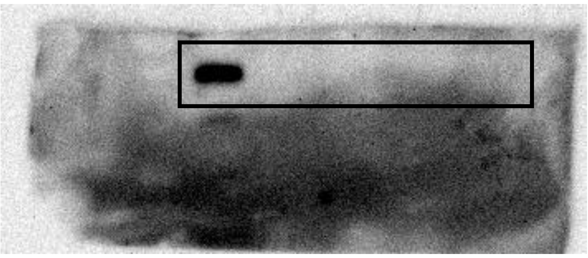

β-actin:

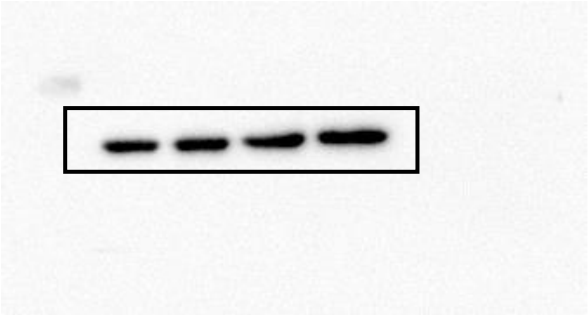

Supplementary Figure 1f:

Nsun6:

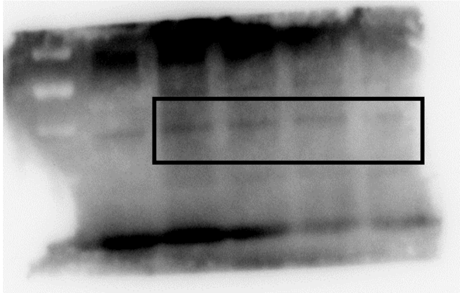

β-actin:

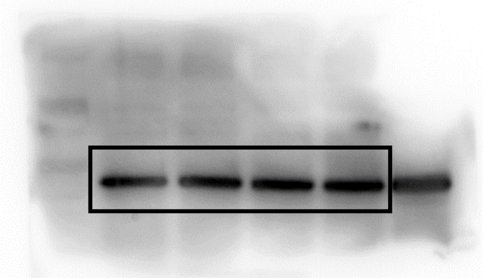

Supplementary Figure 1g:

Mettl3:

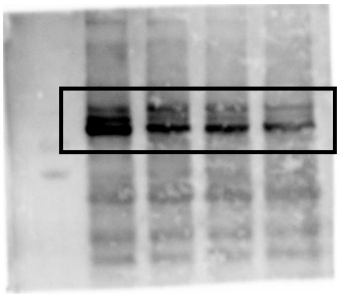

β-actin:

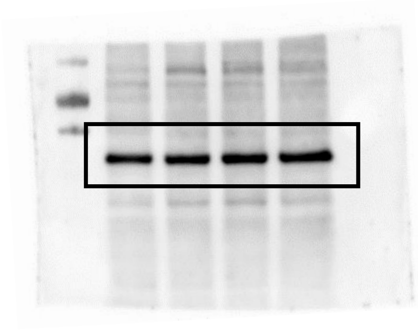

Supplementary Figure 1h:

m^{5}C:

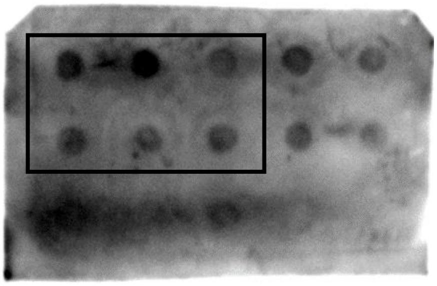

Supplementary Figure 1i:

m^{6}A:

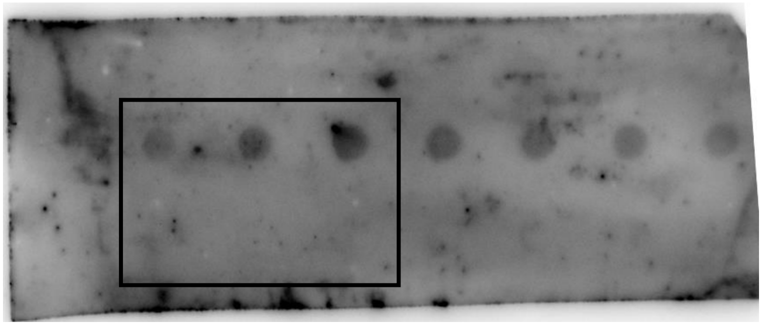

Original scans Supplementary Figure 3

Supplementary Figure 3a:

Mettl3:

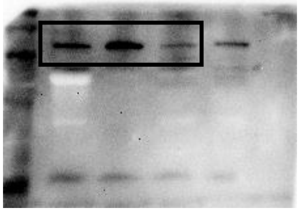

β-actin:

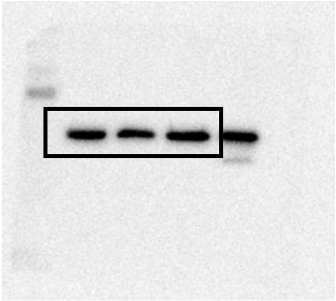

Supplementary Figure 3b:

Nsun4:

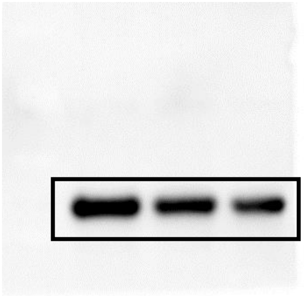

β-actin:

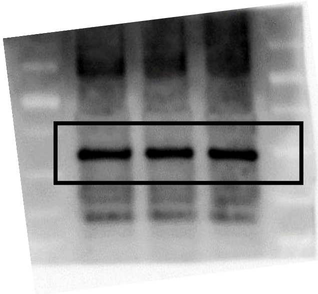

Supplementary Figure 3c:

Mettl3:

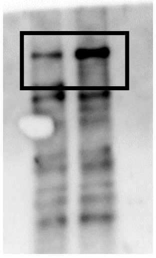

β-actin:

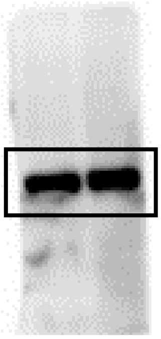

Supplementary Figure 3d:

Nsun4:

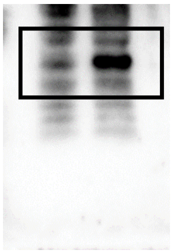

β-actin:

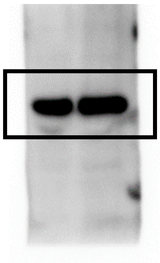

Supplementary Figure 3e:

Mettl3:

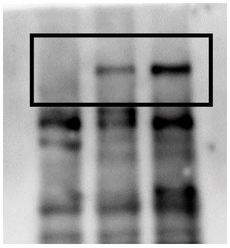

β-actin:

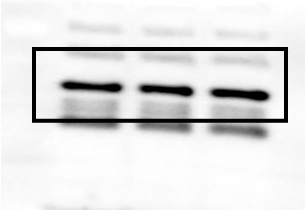

Supplementary Figure 3f:

Nsun4:

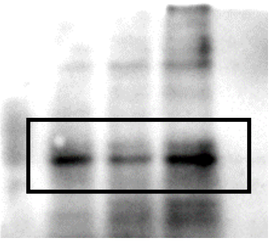

β-actin:

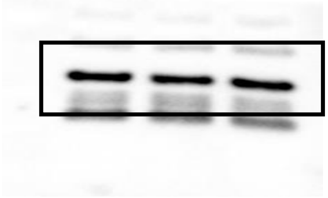

Supplementary Figure 3g-h:

m^5C:

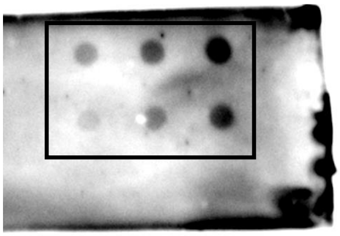

m^6A:

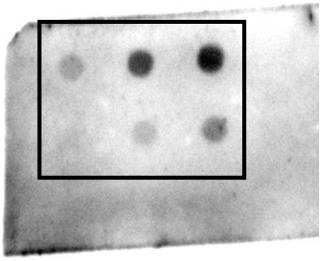

Supplementary Figure 3i-j:

m^6A:

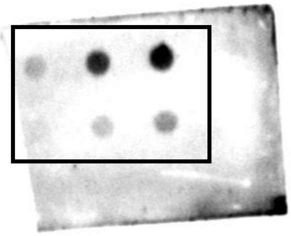

m^5C:

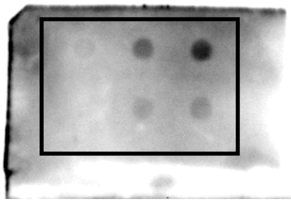

Supplementary Table

Supplementary Table 1. Primer sequences

Primers of Real-time PCR		
Sox9	Forward	TCAACGGCTCCAGCAAGAACAAG
	Reverse	CTCCGCCTCCTCCACGAAGG
Aggrecan	Forward	CTGATCCACTGTCCAAGCACCATG
	Reverse	ATCCACGCCAGGCTCCACTC
Col2	Forward	ACGCTCAAGTCGCTGAACAACC
	Reverse	ATCCAGTAGTCTCCGCTCTTCCAC
Mettl3	Forward	CACGCTGCCTCAGATGTTGACC
	Reverse	CTGACCTTCTTGCTCTGCTGTTCC
Nsun2	Forward	CGCATCCAGCATTCCTAGACTCAC
	Reverse	CGCCACTGCAAGGGACATCAC
Nsun3	Forward	CTTGCTCTTGCGGGGCTATCAC
	Reverse	TCGGTCTGGAGTTCGGCTGAG
Nsun4	Forward	CATGTACCACGGACCGCCATTC
	Reverse	CAAGGAGTCCAGCCGCAAGAAG
Nsun5	Forward	TCACTGGCGGCTTCTTCATTGC
	Reverse	TTGGTGCTTTGCTGAGAGGTTCTG
Nsun6	Forward	AGGAACCACAGATCGGAGGAGAAG
	Reverse	AGGCACAACACACGGGTCAAAG
Nsun7	Forward	CCAATGACGCAGTGACCATACCAG
	Reverse	CAATGCCGCAGCCAACTTTGTC
β-actin	Forward	TGTCACCAACTGGGACGATA
	Reverse	GGGGTGTTGAAGGTCTCAAA
Primers of RIP-qPCR		
Sox9	Forward	TCAACGGCTCCAGCAAGAACAAG
	Reverse	CTCCGCCTCCTCCACGAAGG
Sox9 3'UTR	Forward	GGAAAGCCAAGGGCAAGGA
	Reverse	CAGGCAACCAGGGGCAAAT
Sox9 m6A site:	Forward	CTTGAAGAGCAATGGTGACAGAGTTGATC
	Reverse	TTGCATGAGCTCAGATAATGTCTTAAA
Primers of Bisulfite sequencing		
Sox9 3'UTR Normal primer	Forward	AGCTCACCAGACCCTGAGGAGACCTTGAAGAG
	Reverse	TTCCTCACTGACGCTGGTGGGTCCATCTGGC
Sox9 3'UTR Specific primer	Forward	AGTTTATTAGATTTTGAGGAGATTTTGAAGAG
	Reverse	TTCCTCACTAACRCTAATAAATCCATCTAAC (R=G/A)
28S Normal primer	Forward	GGGGCCTCACGATCCTTCTGACCTTTTGGG
	Reverse	CCAGCTCACGTTCCCTATTAGTGGGTGAAC
28S Specific primer	Forward	GGGGTTTTAYGATTTTTTTGATTTTTTGGG (Y=C/T)
	Reverse	CCAACTCACRTTCCCTATTAATAAATAAAC

Supplementary Table 2. siRNA sequences

siRNAs uesd for this study	
eEF1α-1 siRNAs #1	GAGACTTCATCAAGAACAT
eEF1α-1 siRNAs #2	AGGAAGTCAGCACCTACAT
eEF1α-1 siRNAs #3	GGAAACTGGTGTTCTCAAA
Ythdf2 siRNAs #1	GGGATTGACTTCTCAGCAT
Ythdf2 siRNAs #2	GGGCTGATATTGCTAGCAA
Ythdf2 siRNAs #3	GGTTCTGGATCTACTCCTT
Mettl3 siRNAs #1	GTCTATAGTCCCTGAATTAA
Mettl3 siRNAs #2	CCTACAAGATGACGCACAT
Mettl3 siRNAs #3	TCTCTAAACCTAAGAACTT
Nsun4 siRNAs #1	GCAGAAGTATGGTGCACTA
Nsun4 siRNAs #2	GCCTCAAGATATTAGGAAA
Nsun4 siRNAs #3	CATCAAGGTTCAAGTGGAA
Nsun6 siRNAs #1	AGCCAACAAGGATTGTATA
Nsun6 siRNAs #2	GAATGACAGAACCCATATA
Nsun6 siRNAs #3	TCATGTTCTTGATCCTCAA
