# Supplementary material for: Nsun4 and Mettl3 mediated translational reprogramming of Sox9 promotes BMSC chondrogenic differentiation
Source: Commun Biol. 2022 May 25;5:495. doi: 10.1038/s42003-022-03420-x (PMC9133052; doi:10.1038/s42003-022-03420-x)
Supplement: Supplementary file 3 — Description of Additional Supplementary Files [file 42003_2022_3420_MOESM3_ESM.pdf]

# Description of Additional Supplementary Files

**File name:** Supplementary Data 1
**Description:** Containing source data for all other experiments.
