# Supplementary material for: Nsun4 and Mettl3 mediated translational reprogramming of Sox9 promotes BMSC chondrogenic differentiation
Source: Commun Biol. 2022 May 25;5:495. doi: 10.1038/s42003-022-03420-x (PMC9133052; doi:10.1038/s42003-022-03420-x)
Supplement: Supplementary file 5 — Reporting Summary [file 42003_2022_3420_MOESM5_ESM.pdf]

nature research

Corresponding author(s): DBPR:COMMSBIO-21-0506-T

Last updated by author(s): Feb 24, 2022

Reporting Summary

Nature Research wishes to improve the reproducibility of the work that we publish. This form provides structure for consistency and transparency in reporting. For further information on Nature Research policies, see our Editorial Policies and the Editorial Policy Checklist.

Statistics

For all statistical analyses, confirm that the following items are present in the figure legend, table legend, main text, or Methods section.

n/a	Confirmed	
☐	☒	The exact sample size (*n*) for each experimental group/condition, given as a discrete number and unit of measurement
☐	☒	A statement on whether measurements were taken from distinct samples or whether the same sample was measured repeatedly
☐	☒	The statistical test(s) used AND whether they are one- or two-sided<br>*Only common tests should be described solely by name; describe more complex techniques in the Methods section.*
☐	☒	A description of all covariates tested
☐	☒	A description of any assumptions or corrections, such as tests of normality and adjustment for multiple comparisons
☐	☒	A full description of the statistical parameters including central tendency (e.g. means) or other basic estimates (e.g. regression coefficient) AND variation (e.g. standard deviation) or associated estimates of uncertainty (e.g. confidence intervals)
☐	☒	For null hypothesis testing, the test statistic (e.g. F, t, r) with confidence intervals, effect sizes, degrees of freedom and P value noted<br>*Give P values as exact values whenever suitable.*
☒	☐	For Bayesian analysis, information on the choice of priors and Markov chain Monte Carlo settings
☒	☐	For hierarchical and complex designs, identification of the appropriate level for tests and full reporting of outcomes
☒	☐	Estimates of effect sizes (e.g. Cohen's *d*, Pearson's *r*), indicating how they were calculated

Our web collection on statistics for biologists contains articles on many of the points above.

Software and code

Policy information about availability of computer code

Data collection	no software were used for data collection
Data analysis	Graphpad Prism 7.00 was used to statistical analysis

For manuscripts utilizing custom algorithms or software that are central to the research but not yet described in published literature, software must be made available to editors and reviewers. We strongly encourage code deposition in a community repository (e.g. GitHub). See the Nature Research guidelines for submitting code & software for further information.

Data

Policy information about availability of data

All manuscripts must include a data availability statement. This statement should provide the following information, where applicable:

- Accession codes, unique identifiers, or web links for publicly available datasets
- A list of figures that have associated raw data
- A description of any restrictions on data availability

The authors declare that the data supporting the findings of this study is provided in the form of figures. Raw data is also submitted as supplementary data file and available from the corresponding author upon reasonable request.

Field-specific reporting

Please select the one below that is the best fit for your research. If you are not sure, read the appropriate sections before making your selection.

☒ Life sciences ☐ Behavioural & social sciences ☐ Ecological, evolutionary & environmental sciences

For a reference copy of the document with all sections, see nature.com/documents/nr-reporting-summary-flat.pdf

Life sciences study design

All studies must disclose on these points even when the disclosure is negative.

Sample size	No sample size estimation was performed.
Data exclusions	No data were excluded from the analysis.
Replication	Repeated 2-6 times.
Randomization	For the animal study, all animals within the four experimental groups were delivered within the same week and derived from the same breeding cage.
Blinding	N/A

Reporting for specific materials, systems and methods

We require information from authors about some types of materials, experimental systems and methods used in many studies. Here, indicate whether each material, system or method listed is relevant to your study. If you are not sure if a list item applies to your research, read the appropriate section before selecting a response.

Materials & experimental systems

n/a	Involved in the study	
☐	☒	Antibodies
☐	☒	Eukaryotic cell lines
☒	☐	Palaeontology and archaeology
☐	☒	Animals and other organisms
☒	☐	Human research participants
☒	☐	Clinical data
☒	☐	Dual use research of concern

Methods

n/a	Involved in the study	
☒	☐	ChIP-seq
☒	☐	Flow cytometry
☒	☐	MRI-based neuroimaging

Antibodies

Antibodies used	Anti-rabbit Nsun4 (Novus, cat#NBP2-19594, lot 40380); Anti-rabbit Nsun6 (protein tech, cat# 17240-1-AP, lot 8598); Anti-rabbit Mettl3 (protein tech, cat# 15073-1-AP, lot 93976); Anti-rabbit Sox9 (abcam, cat# ab185966, lot GR3241181-1); anti-mouse Aggrecan (abcam, cat# ab3778, lot GR3235547-1); anti-mouse Col2 (abcam, cat# ab185430, lot GR3250036-1); anti-rabbit m6A (SYSY, cat# 202 003, lot 2-94); anti-mouse m5C (abcam, cat# ab10805, lot GR300572-9); anti-rabbit Ythdf1 (protein tech, cat# ab157542, lot GR3227512-6); anti-rabbit Ythdf2 (Millipore, cat# ABE542, lot 3170165); anti-rabbit Ythdf3 (protein tech, cat# ab83716, lot GR3203031-7); anti-rabbit eEF1a-1 (protein tech, cat# 11402-1-AP, lot 53965); Anti-beta Actin (abcam, cat# ab49900, lot GR3184659-16); anti-mouse IgG H&L (Alexa Fluor® 647) (abcam, cat# ab150115, lot GR3227129-2) anti-rabbit IgG H&L (Alexa Fluor® 647) (abcam, cat# ab150075, lot GR3312062-1).
Validation	All antibodies used in this study have been extensively validated in numerous published studies or in commercial websites.A

Eukaryotic cell lines

Policy information about cell lines

Cell line source(s)	Hek293T cell lines used in this study were purchased from TAKARA (Tokyo, Japan).
Authentication	N/A
Mycoplasma contamination	All cell lines used in this study were tested and found negative for Mycoplasma contamination.
Commonly misidentified lines (See ICLAC register)	N/A

Animals and other organisms

Policy information about studies involving animals; ARRIVE guidelines recommended for reporting animal research

Laboratory animals	Male Sprague-Dawley (SD) rats averaging 190-210 g in weight were used in this study.
Wild animals	N/A
Field-collected samples	Housing in Guangdong Medical University.
Ethics oversight	All animal experimental procedures were approved by the Experimental Ethics Committee of Guangdong Medical University.

Note that full information on the approval of the study protocol must also be provided in the manuscript.
